# Biofilmed multifarious rhizobacterial isolates of tomato rhizosphere of North-Western Himalayas promote plant growth in tomato

**DOI:** 10.3389/fpls.2025.1610707

**Published:** 2025-07-01

**Authors:** Shubham Kaundal, Neerja Rana, Yashwant Kumar, Saleh S. Alhewairini, Jayanthi Barasarathi, Farah Farhanah Haron, Nazih Y. Rebouh

**Affiliations:** ^1^ Department of Basic Sciences, College of Forestry, Dr. Yashwant Singh Parmar University of Horticulture and Forestry, Solan, India; ^2^ Central Research Institute Kasauli, Kasauli, Solan, India; ^3^ Department of Plant Protection, College of Agriculture and Food, Qassim University, Qassim, Saudi Arabia; ^4^ Faculty of Health & Life Sciences (FHLS), INTI International University, Nilai, Negeri Sembilan, Malaysia; ^5^ Agrobiodiversity and Environment Research Centre, Malaysian Agricultural Research and Development Institute (MARDI), MARDI Headquarters, Serdang, Selangor, Malaysia; ^6^ Department of Environmental Management, Institute of Environmental Engineering, RUDN University, Moscow, Russia

**Keywords:** ammonia, biofilm-producing, HCN, indole acetic acid, PGPR, P solubilization

## Abstract

**Background:**

Tomato production is often limited by poor soil health and nutrient deficits, which lower agricultural productivity. Plant growth-promoting rhizobacteria (PGPR) provide a sustainable approach to improve plant development and soil fertility.

**Objectives:**

The objectives of this study were to 1) isolate and screen PGPR from the rhizosphere soil of tomato-growing regions in Himachal Pradesh, India; 2) evaluate the selected PGPR for biofilm production; 3) characterize and molecularly identify the biofilm-producing isolates; and 4) assess their efficacy in enhancing tomato plant growth.

**Methods:**

Forty bacterial isolates were collected from soils in Dharon Ki Dhar, Shillai, Balh, and Berthin and tested for PGPR characteristics. These included phosphate solubilization, nitrogen fixation, and the production of hydrogen cyanide, ammonia, and indole-3-acetic acid (IAA). Siderophore production and biofilm formation were also assessed. The most potent biofilm-producing isolates were identified using 16S rDNA sequencing.

**Results:**

Among the isolates, 28 solubilized phosphate (up to 91.2% with MB-7), 26 fixed nitrogen, 18 produced hydrogen cyanide, and 16 produced ammonia. All isolates produced IAA, with MB-7 and BB-3 producing the highest quantities (89.1 and 85.1 µg/mL, respectively). BB-3 exhibited the highest percentage of siderophore production (86.2%). BB-3 and MB-7 were potent biofilm producers. Molecular analysis identified BB-3 as *Brucella rhizosphaerae* and MB-7 as *Delftia lacustris*. Inoculation with *D. lacustris* greatly enhanced tomato plant growth—plant height increased by 49.14%, shoot fresh weight increased by 32.47%, and root length increased by 45.00%—as compared to uninoculated control.

**Conclusion:**

*D. lacustris* shows significant potential as a bioinoculant for increasing tomato plant growth and can potentially be used effectively in sustainable agriculture approaches.

## Introduction

The global reliance on chemical fertilizers has prompted severe environmental concerns due to their detrimental effects on soil health and fertility (Jabborova et al., 2021; Khan et al., 2025). Excessive mineral fertilizer application pollutes the environment and degrades agricultural soils over time. Biofertilizers have been developed as environmentally acceptable options for increasing the growth of crops while maintaining soil quality (Sayyed et al., 2018; Jabborova et al., 2025; Najafi et al., 2021). Despite significant scientific advances, the use of microbial inoculants in agriculture remains low (Saranraj et al., 2023). Microbial inoculants, particularly plant growth-promoting rhizobacteria (PGPR), provide promising solutions by increasing nutrient availability (Sethi et al., 2025); synthesizing phytohormones such as indole-3-acetic acid (IAA), cytokinins, and gibberellins (Bright et al., 2025); and inducing systemic resistance to pathogens (Praveen et al., 2024; Dave et al., 2024; Omar et al., 2022; Vinothini et al., 2024). Additionally, PGPR reduce drought stress in plants (Dhiman et al., 2024).

One of the major drawbacks of using free-cell microbial biofertilizers is their low survival rate in soil (Malusa et al., 2012). This difficulty can be solved using biofilm-forming PGPR, which are more resistant to environmental challenges (Ferioun et al., 2025). Biofilms are microbial communities enclosed in an extracellular matrix made up of exopolysaccharides, proteins, lipids, and DNA (Qurashi and Sabri, 2012; Ilyas et al., 2020; Bright et al., 2025). These structures outperform planktonic bacteria in terms of cell survival, root colonization, nutrient uptake, and pathogen suppression (Backer et al., 2018; Budamagunta et al., 2023).

Beneficial biofilm-forming PGPR, such as *Bacillus*, *Pseudomonas*, and *Enterobacter*, are essential for nutrient solubilization, stress reduction, and soil structure improvement (Gogoi et al., 2021; Ajijah et al., 2023). Their resistance to extreme environmental circumstances makes them ideal for sustainable agriculture, particularly in saline- and nutrient-poor soils (Haroon et al., 2023; Ansari et al., 2021; Brokate et al., 2024). However, only a few biofilm-producing strains have been identified, and more study is needed to investigate their potential in field conditions (Mahmood et al., 2016; Gupta et al., 2017).

Tomato (*Solanum lycopersicum* L.), the world’s second most extensively produced vegetable, is high in antioxidants like lycopene, phenolics, and micronutrients (Irina et al., 2024). Its production is under threat from environmental challenges such as drought and exploitation of chemical inputs (Alkilayh et al., 2024; Yatoo et al., 2024). In this context, biofilm-forming PGPR represent a viable technique for improving tomato growth and resilience while lowering reliance on agrochemicals (Bright et al., 2025).

## Methods

### Collection of soil samples

Four soil samples were collected from tomato-growing areas in Himachal Pradesh, including Dharon Ki Dhar (30.8813°N, 77.1775°E) in District Solan, Shillai (30.6748°N, 77.7066°E) in District Sirmaur, Balh (31.6079°N, 76.9129°E) in District Mandi, and Berthin (31.4188°N, 76.6427°E) in District Bilaspur, India. Soil samples were taken from the rhizosphere (15–20-cm depth) of tomato plants in moderate climatic conditions between mid-March and June 2022. Only one soil sample was taken from each field/rhizosphere. Average temperatures ranged from 10.6°C in March to 20.6°C in June, with relative humidity rising from 50%–60% to approximately 64%. Shillai, being more elevated, had slightly cooler conditions. Rainfall varied by month, peaking at 167 mm in June. The above conditions were favorable for microbial activity and rhizospheric interactions. The samples were placed in sterile plastic bags and stored in the laboratory until they were ready for further examination.

### Physicochemical, available nutrient, and microbiological properties of soil mixture

The collected soil samples were analyzed for important physicochemical, available nutrient status, and microbiological properties by adopting the following standard procedures.

#### pH

The soil pH was determined in 1:2.5 soil:water suspension using the method described by Jackson (1973). The soil was mixed with distilled water in a 1:2.5 ratio to prepare a suspension. A calibrated pH meter was used to measure the pH, ensuring that the electrode remained submerged without touching the beaker walls. The pH of the diluted soil mixture was recorded.

#### Organic carbon

Organic carbon was determined using the chromic acid titration method of Walkley and Black (1934). In this method, 1.00 g of processed soil was weighed, and 10 mL of potassium dichromate and 20 mL of sulfuric acid were added. After 5 min, the mixture was diluted with deionized water to 125 mL, mixed, and allowed to cool. Five to six drops of ferroin indicator were added, and then the mixture was titrated with ferrous sulfate, recording the volume to the nearest 0.1 mL. Blank samples were titrated before unknown samples, ensuring that the titrant volume was within 0.2 mL.

#### Available nitrogen

Available nitrogen was determined using the alkaline permanganate method of Subbiah (1956). A 20-g soil subsample (<2 mm) was placed in an 800-mL Kjeldahl digestion flask with 100 mL of 0.32% KMnO_4_, 100 mL of 2.5% NaOH, and 20 mL of water. The flask was connected to a macro-Kjeldahl distillation unit, and 75 mL of distillate was collected in 25 mL of boric acid indicator mixture. The ammonia was titrated with 0.05 N H_2_SO_4_ to determine the available nitrogen content.

#### Available phosphorus

The P content of soil was determined according to the method of Olsen (1954). Briefly, 0.5 N sodium bicarbonate (NaHCO_3_) at pH 8.5 was used to extract available phosphorus, and the resulting solution was subjected to P estimation using a spectrophotometer.

#### Available potassium

Available potassium was extracted using neutral normal ammonium acetate (Merwin and Peech, 1951) and determined on a flame photometer. Plant-available potassium was measured by analyzing the filtered extract on an atomic absorption spectrometer set to emission mode at 766.5 nm. The results were reported as parts per million (ppm) of potassium (K) in the soil.

#### Total viable bacterial count

The soil samples were analyzed for viable bacterial count before the experiment. One gram of soil in 9 mL of sterilized water blank and the soil suspension was diluted in a 10-fold series, and then bacterial count was determined using the standard pour plate technique on different media as described by Subba Rao (1999). The count was expressed as colony-forming units per gram of soil (cfu/g soil).

#### Isolation and screening of bacteria for plant growth-promoting metabolites

The biofilm-producing bacteria were isolated from different soil samples by serial dilution and spread plate techniques. The serially diluted suspensions of samples were spread on a pre-poured nutrient agar medium. The plates were incubated for 24 h at 30°C ± 2°C. The pure culture of bacteria was obtained by repeated sub-culturing. The isolates were further screened for plant growth-promoting (PGP) traits such as phosphate solubilization, nitrogen-fixing ability, IAA production, HCN production, ammonia production, and siderophore production.

#### Screening for phosphate solubilization

The phosphorus-solubilizing activity of isolates was determined qualitatively. The isolates were spot-inoculated on Pikovskaya’s Medium (PVK) medium as described by Pikovskaya (1948) and incubated at 30°C ± 2°C for 72 h. The yellow-colored halo zones around the bacterial colony indicate the phosphate-solubilizing activity of the bacterial isolates. The halo zone diameter around the colony was calculated by subtracting the colony size from the total halo zone size. The phosphate solubilization index (PSI) was measured using the following formula (Edi-Premono et al., 1996):

#### Screening for nitrogen fixation

Bacterial isolates were spot-inoculated on Jensen’s medium and incubated at 28°C ± 2°C for 96 h. The plates that showed growth in the form of a colony were selected (Jensen, 1954).

#### Screening for indole-3-acetic acid production

Bacterial isolates were inoculated in a nutrient broth enriched with tryptophan (1%–2%) and incubated for 24 h at 28°C ± 2°C under shaking conditions. Cultures were centrifuged at 15,000 rpm for 20 min. Following the centrifugation, 2 mL of Salkowski reagent was added to the supernatant, incubated for 30 min at 28°C ± 2°C, and observed for the development of pink color as an indication of IAA production. Quantitative estimation of IAA from supernatant was performed using the method of Gordon and Paleg (1957).

#### Screening for HCN production

Bacterial isolates were screened out for the production of hydrogen cyanide (HCN) as per the method described by Bakker and Schippers (1987). Bacterial cultures were streaked on pre-poured plates of King’s B medium amended with 1.4 g/L glycine. Whatman No. 1 filter paper strips were soaked in 0.5% picric acid in 2% sodium carbonate and placed in the lid of each Petri plate. The plates were sealed with parafilm and incubated at 30°C ± 2°C for 1–4 days. Uninoculated control was kept for comparison of results. Plates were observed for change in color of filter paper from yellow to orange-brown.

#### Screening for ammonia production

Each of the bacterial isolates was cultured in peptone water at 30°C ± 2°C for 4 days. One milliliter of Nessler’s reagent was added to each tube. The development of a yellow-to-orange color indicated the production of ammonium by the bacterial isolates (Ahmad et al., 2008).

#### Screening for siderophore production

Siderophore production was detected using the chrome azurol S (CAS) plate assay method (Patel et al., 2018; Schwyn and Neilands, 1987). Sterilized blue agar was prepared by mixing CAS (60.5 mg/50 mL distilled water) with 10 mL of iron solution (1 mM FeCl_3_·6H_2_O in 10 mM HCl). This solution was slowly added to a hexadecyltrimethyl ammonium bromide (HDTMA) solution prepared by dissolving 72.9 mg HDTMA in 40 mL distilled water. Thus, 100 mL of CAS dye was prepared; 750 mL of nutrient agar was mixed with 1,4-piperazine diethane sulfonic acid (30.24 g), and pH 6.8 was adjusted with 0.1 N NaOH. It was autoclaved separately and then mixed with CAS (100 mL) under aseptic conditions, and then the plates were prepared for further experiments. A bit of 72-h-old culture of each test bacterium was placed on pre-poured blue-colored CAS agar plates. Plates were incubated at 30°C ± 2°C for 24 h and observed for the production of an orange halo around the bit.


$$\text{SE}(\%)=\frac{Z-C}{C}\times 100$$

where SE is the siderophore efficiency (%), Z is the halo zone diameter (mm), and C is the colony diameter (mm).

#### Screening of selected PGP isolates for biofilm formation

The selected PGP isolates were then tested for biofilm production by inoculating bacteria freshly isolated from agar plates into 10 mL of trypticase soy broth (TSB) with 1% glucose (Christensen et al., 1985). The broths were incubated at 37°C ± 2°C for 24 h and then diluted with 1:100 fresh medium; 200 µL of diluted cultures was placed in each well of a sterile 96-well flat-bottom polystyrene tissue culture plate. The negative control wells contained uninoculated sterile broth. The plates were incubated at 37°C ± 2°C for another 24 h. After incubation, the contents of each well were gently tapped out and washed four times with 0.2 mL of phosphate-buffered saline (PBS; pH 7.2) to remove any free-floating bacteria. Biofilms formed by bacteria adhering to the well walls were fixed with 2% sodium acetate and stained with 0.1% crystal violet. The excess stain was removed by washing with deionized water, and the plates were left to dry. After drying, the wells were filled with 99.9% isopropyl alcohol (IPA) to dissolve the crystal violet. The optical density (OD) of the stained adherent biofilm was measured at 570 nm using a micro-ELISA auto reader. The experiment was conducted in triplicate and repeated three times. Biofilm production was assessed using the criteria established by Stepanovic et al. (2007).

#### Phenotypic characterization of isolates

Standard biochemical assays were used to evaluate the phenotypic characteristics of rhizobacterial isolates. Catalase activity was measured by observing the formation of oxygen bubbles in pure cultures after adding 3% H_2_O_2_ (Graham and Parker, 1964). Gram staining was conducted on log-phase cultures and viewed using a phase-contrast microscope, as described by Coico (2006). Citrate utilization was assessed on Simmons citrate agar, with the appearance of a transparent zone indicating positive utilization (Koser, 1924). Casein hydrolysis was examined on skim milk agar plates, and clear zones formed, indicating protease activity (Wehr and Frank, 2004; Jadhav et al., 2020). All tests included uninoculated controls.

### Molecular characterization of bacterial isolates

#### Isolation of genomic DNA

Bacterial genomic DNA was isolated by taking a 24-h-old broth culture (10 mL) of bacterial isolates. The broth culture was centrifuged at 2,000 rpm for 10 min, and the supernatant was discarded. One milliliter of freshly prepared extraction buffer was added to the pellet, the resulting solution was transferred to a 2-mL Eppendorf tube and incubated at 65°C in a water bath for 30 min, and an equal volume of an ice-cold solution of phenol:chloroform:isoamyl alcohol (25:24:1) was added to it. The solution was mixed well and centrifuged at 10,000 rpm for 10 min at 4°C, and the upper aqueous layer was transferred to a new Eppendorf tube. An equal volume of an ice-cold solution of phenol:chloroform:isoamyl alcohol (25:24:1) was added to this aqueous layer, and the step was repeated three to four times. In the final aqueous layer, the ice-cold absolute alcohol was added in excess to precipitate the bacterial genomic DNA, and the solution was centrifuged at 10,000 rpm at 4°C for 10 min. The supernatant was discarded, the pellet was washed with 100 µL of 70% (v/v) ethanol and centrifuged at 10,000 rpm for 15 min at 4°C, the supernatant was discarded, and the pellet was dissolved in 50 µL of Tris-EDTA (TE) buffer (pH 8.0) and stored at −20°C for further studies (Sambrook et al., 1989).

#### Amplification and sequencing of 16S rDNA

Universal primers were applied to amplify the 16S rDNA sequences of the biofilm-producing PGP isolates. To amplify the bacterial 16S rDNA gene, a conserved ~1.5-kb region was targeted with the following primer pair:

Forward primer (16S-F): 5′-GGATGAGCCCGCGGCCTA-3′.

Reverse primer (16S-R): 5′-CGGTGTGTACAAGGCCCGG-3′.

These primers were chosen for their high specificity and Guanine-Cytosine (GC) content (72.22% for forward and 68.42% for reverse), and they were designed to assure efficient and accurate amplification with high-fidelity DNA polymerase. The primer pair amplifies a region that is useful for taxonomic identification and phylogenetic analysis of bacterial isolates.

The PCR was carried out in a 20-µL reaction containing ~50 ng of template DNA, 20 pM of each primer, 0.2 mM dNTPs, and 1 U Taq polymerase (Genei, Bangalore, India) in 1× PCR buffer. Amplification was performed using a thermal cycler (multigene PCR system, Labnet, Edison, NJ, USA). Reactions were cycled 35 times at 94°C for 3 min, 50°C for 30 sec, and 72°C for 1.5 min followed by a final extension at 72°C for 10 min. The PCR products were analyzed on 1% agarose gel in 1× Tris-Acetate-EDTA (TAE) buffer and ran at 100 V for 1 h, and the size was determined using a 1-kb DNA ladder (Thermo Scientific, Waltham, MA, USA). The amplified product was purified and sequenced by Biokart Genomics Lab, Bengaluru, India. The sequences were aligned using ClustalW 1.74 followed by the construction of a neighbor-joining phylogenetic tree using MEGA11 (Tamura et al., 2013). The sequences were aligned using ClustalW 1.74, and a neighbor-joining phylogenetic tree was constructed using MEGA11 (Tamura et al., 2013).

### Plant growth promotion studies

#### Tomato seeds

The seeds of the tomato (cultivar ‘Solan Lalima’) were procured from the Department of Seed Science and Technology, Dr. YS Parmar University of Horticulture and Forestry, Nauni-Solan, India. This cultivar was chosen for the study, as it is a local and commonly grown tomato variety in the area and is also very responsive to the inoculation of biofertilizers.

#### Inoculum preparation

For the preparation of the inoculum, biofilm-producing PGP bacterial isolates were cultured in a liquid medium. The medium was incubated at 28°C for 48 h. The population density (1.5 OD at 540 nm) that resulted in the formation of 10^8^ cfu/mL of the bacterial isolate was used for the preparation of inoculum formulation.

#### Seed inoculation and nursery preparation

The selected seeds of the cultivar ‘Solan Lalima’ were immersed in a sterile beaker for 1 h in a liquid culture of various bacterial formulations. The seeds were inoculated with selected bacterial isolates, and the nursery was produced by transferring the seeds into disposable cups. Following germination, the seedlings were planted into pots. One uninoculated control treatment was kept for comparison. The pots were arranged in triplicate, with each treatment having three replications, and placed under net house conditions.

#### Preparation of potting mixture

The soil was obtained from a furrow slice (0- to 15-cm depth) of the soil science field, UHF Nauni Solan. The soil was passed through a 2-mm sieve and used for a pot culture experiment. The potting mixture was prepared by mixing sand, soil, and farm yard manure (FYM) in a ratio of 1:2:1. The mixture was sterilized for three successive autoclave cycles of 1 h each at 121°C. Five kilograms of potting mixture was filled in each pot. Moisture in the potting mixture was maintained near field capacity.

### Measurement of plant growth parameters

#### Plant height (cm)

Plant height was measured from the soil base to the tip of the fully expanded leaf, and values were recorded in centimeters.

#### Shoot fresh weight (g)

The shoot of the plants was weighed separately immediately after harvesting, and values were expressed in grams.

#### Root length (cm)

The length of roots was measured using a scale for individual plants and expressed in centimeters.

### Statistical analysis

Statistical analysis was performed using the OPSTAT software to establish the significance of the treatments and mean, as well as to draw appropriate conclusions. Data on the physicochemical parameters of various soil samples collected were statistically analyzed using a suitable analysis of variance method based on one-factor completely randomized design (CRD) analysis. The significance of the treatment effect was determined using the “F” test (variance ratio). The difference in treatment means was assessed using a critical difference (CD) at a 5% level of probability (Gomez and Gomez, 1984). If the test’s variance ratio was significant at the 0.05% level, the standard error of the mean (S.Em.±) and CD were determined for treatment comparisons.

## Results

### Physicochemical properties of the samples collected from tomato-growing fields

**Table 1** shows significant variations in the soil properties of the samples collected from different locations. The data revealed that there was no significant variation in the soil pH from different locations. However, the organic carbon (OC) and available N, P, and K varied significantly. The maximum values for OC (1.38%), available N (356.25 kg/ha), and available P (55.35 kg/ha) were recorded in the Balh site of District Mandi, and the available K was found to be maximum (310.15 kg/ha) in the Shillai site of District Sirmaur. However, the minimum values for OC (0.94%) and available P (30.31 kg/ha) were recorded in the Berthin site of District Bilaspur, and the available N and K were found to be minimum (287.63 and 211.42 kg/ha, respectively) in the Dharon Ki Dhar site of District Solan.

**Table 1:** Physicochemical properties of the soil samples collected from tomato-growing areas of Himachal Pradesh.

District	Site	pH	Organic carbon (%)	Available N (kg/ha)	Available P (kg/ha)	Available K (kg/ha)
Solan	Dharon Ki Dhar	6.99 ± 0.02	1.18 ± 0.02	287.63 ± 0.02	47.13 ± 0.02	211.42 ± 0.02
Sirmaur	Shillai	6.92 ± 0.02	1.25 ± 0.04	302.78 ± 0.03	35.62 ± 0.04	310.15 ± 0.07
Mandi	Balh	6.85 ± 0.03	1.38 ± 0.05	356.25 ± 0.04	55.35 ± 0.05	233.82 ± 0.08
Bilaspur	Berthin	6.67 ± 0.04	0.94 ± 0.03	298.45 ± 0.03	30.31 ± 0.04	285.98 ± 0.04
**S.Em.**		0.11	0.02	3.96	0.79	3.41
**CD _(0.05)_**		NS	0.07	12.94	1.53	11.13

Values are the average of triplicates analyzed by ANOVA. ± indicates standard deviation. The significance of the effect was judged with the help of the “F” test. The difference in the mean values was tested using critical difference (CD) at a 5% level of probability. The variance ratio of the test at 0.05% level of significance was further subjected to S.Em.± and CD.

### Isolation of bacteria from tomato-growing fields

In the present study, a total of four soil samples from tomato-growing areas of Himachal Pradesh, India, were screened for the presence of biofilm-producing multifarious PGPR. A total of 74 bacterial isolates were isolated from four soil samples collected from tomato-growing areas of Himachal Pradesh: 20 isolates (SD-1–SD-20) were isolated from the Dharon Ki Dhar site of District Solan, 14 isolates (SS-1–SS-14) were isolated from the Shillai site of District Sirmaur, 20 isolates (MB-1–MB-20) were isolated from the Balh site of District Mandi, and 20 isolates (BB-1–BB-20) were isolated from the Berthin site of District Bilaspur. The bacterial count of the isolates was also recorded to range from 5.83 to 6.42 (×10^6^ log cfu/g). The maximum count was recorded in site Balh (6.42 × 10^6^ log cfu/g) of District Mandi and the minimum count in site Berthin (5.83 × 10^6^ log cfu/g) of District Bilaspur (**Table 2**).

**Table 2:** Enumeration of Total Viable Count (TVC) of bacterial isolates from tomato-growing fields.

District	Site	Bacterial count (×10^6^ log cfu/g)
Solan	Dharon Ki Dhar	6.31 ± 0.01
Sirmaur	Shillai	6.24 ± 0.01
Mandi	Balh	6.42 ± 0.02
Bilaspur	Berthin	5.83 ± 0.03

Values are the average of triplicates analyzed by ANOVA. ± indicates standard deviation.

### Screening of bacterial isolates for PGP traits

**Figure 1:**
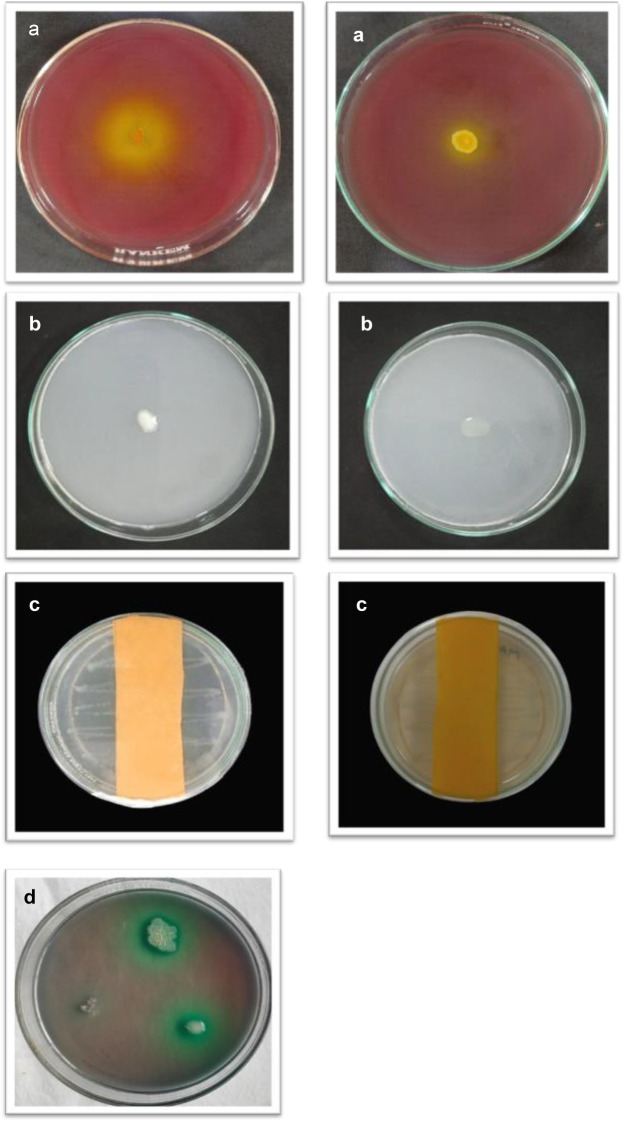
**(a)** Phosphate solubilization. **(b)** Nitrogen fixation. **(c)** HCN production. **(d)** Siderophore production.

A total of 40 isolates showed positive results for PGP activities such as phosphate solubilization, nitrogen-fixing ability, IAA, HCN, ammonia, and siderophore production (**Figure 1**). All the isolates produced IAA, with the maximum produced by MB-7 (89.1 µg/mL) followed by BB-3 (85.1 µg/mL) (**Table 3**). Phosphate solubilization was demonstrated by 28 bacterial isolates. The maximum phosphate solubilization efficiency was observed in MB-7 (91.2%) followed by isolate BB-3 (86.2%). The 26 isolates showed growth in nitrogen-free Jensen’s media, 18 bacterial isolates showed HCN production by completely changing the color of filter paper from yellow to dark brown; 16 isolates showed ammonia production by developing a brown-to-yellow color with the addition of Nessler’s reagent, and only 15 isolates showed positive results for siderophore production. The maximum siderophore production efficiency was observed in isolate BB-3 (86.2%) followed by MB-7 (82.6%).

**Table 3:** Screening of bacterial isolates for multifarious plant growth-promoting traits.

Bacterial isolates	P solubilization (% Phosphate Solubilization Efficiency (PSE))	P production in liquid medium (µg/mL)	Nitrogen fixation	IAA production (µg/mL)	HCN production	Ammonia production	Siderophore production (% SE)
SD-3	42.0 ± 0.15	37.5 ± 0.17	+	35.5 ± 0.10	−	++	−
SD-8	52.5 ± 1.89	22.6 ± 0.51	+	15.2 ± 0.10	−	++	14.2 ± 0.62
SD-10	57.6 ± 1.61	15.4 ± 0.68	+	25.3 ± 0.25	−	+	10.6 ± 0.08
SS-1	59.2 ± 0.21	36.1 ± 1.01	−	40.2 ± 1.27	+	+	75.0 ± 1.62
SS-5	29.4 ± 0.58	28.9 ± 1.02	++	68.4 ± 0.80	−	++	47.1 ± 0.85
SS-8	36.1 ± 0.20	20.8 ± 0.11	++	36.5 ± 0.79	+	+++	35.2 ± 0.60
SS-11	41.6 ± 0.26	17.6 ± 0.22	+	39.7 ± 0.21	−	+	25.1 ± 0.84
MB-1	66.6 ± 1.68	35.2 ± 1.52	−	36.2 ± 1.44	−	++	40.0 ± 1.69
MB-3	27.2 ± 0.83	28.6 ± 0.88	−	46.8 ± 1.22	−	++	50.0 ± 0.63
MB-4	60.0 ± 2.38	25.3 ± 0.32	−	56.1 ± 2.12	−	++	28.4 ± 0.46
MB-7	91.2 ± 2.30	84.2 ± 1.06	++	89.1 ± 0.32	+	+	82.6 ± 1.79
BB-3	86.2 ± 2.64	80.8 ± 1.97	+	85.1 ± 1.69	+	+++	86.2 ± 3.34
BB-4	44.2 ± 1.31	47.6 ± 2.06	+	67.3 ± 2.43	−	+++	−
BB-5	71.4 ± 2.77	75.2 ± 0.69	++	47.3 ± 0.81	++	++	74.1 ± 0.87
BB-8	43.6 ± 1.69	19.5 ± 0.16	+	45.2 ± 0.41	−	++	36.4 ± 0.75
S.Em.	0.94	0.59		0.67			0.78
CD _(0.05)_	2.63	1.71		2.29			2.11

The plant growth-promoting (PGP) assays were conducted in replicates, with each value with three replications. Values are the average of triplicates analyzed by ANOVA. ± indicates standard deviation. The significance of the effect was judged with the help of the “F” test. The difference in the mean values was tested using critical difference (CD) at a 5% level of probability. The variance ratio of the test at 0.05% level of significance was further subjected to S.Em.± and CD.

IAA, indole-3-acetic acid.

### Screening of selected PGP isolates for biofilm formation

A total of 15 selected PGP bacterial isolates were further screened for biofilm formation using the tissue culture plate method. The data presented in **Table 4** and **Supplementary Figure 1** revealed a significant variation among 15 bacterial isolates. The maximum average OD value at 570 nm was observed in BB-3 (0.204) followed by MB-7 (0.186). However, the minimum siderophore unit was recorded in SK-8 (0.116). Isolates MB-7 and BB-3 produced higher amounts of Extracellular Polymeric Substances (EPS) compared to others, while the majority of the isolates did not produce biofilm (**Tables 4**, **5**).

**Table 4:** Selection of efficient PGPR for biofilm formation by TCP method.

Bacterial isolates	Average OD value (at 570 nm)	Biofilm production
SD-3	0.056 ± 0.00	Non
SD-8	0.138 ± 0.01	Weak
SD-10	0.042 ± 0.00	Non
SS-1	0.120 ± 0.00	Weak
SS-5	0.052 ± 0.00	Non
SS-8	0.024 ± 0.00	Non
SS-11	0.016 ± 0.00	Non
MB-1	0.112 ± 0.00	Non
MB-3	0.052 ± 0.00	Non
MB-4	0.062 ± 0.00	Weak
MB-7	0.186 ± 0.01	Strong
BB-3	0.204 ± 0.01	Strong
BB-4	0.021 ± 0.00	Non
BB-5	0.126 ± 0.01	Moderate
BB-8	0.031 ± 0.00	Non
S.Em.	0.0018	
CD _(0.05)_	0.0041	

Values are the average of triplicates analyzed by ANOVA. ± indicates standard deviation. The significance of the effect was judged with the help of the “F” test. The difference in the mean values was tested using critical difference (CD) at a 5% level of probability. The variance ratio of the test at 0.05% level of significance was further subjected to S.Em.± and CD.

PGPR, plant growth-promoting rhizobacteria; TCP, tissue culture plate.

**Table 5:** Interpretation of biofilm production.

Average OD value	Biofilm production
≤0.050/0.118< ~ ≤ 0.124	Non/weak
0.124< ~ ≤ 0.174	Moderate
>0.185	Strong

OD, optical density.

### Biochemical tests

Various biochemical tests were performed for biofilm-forming bacterial isolates showing maximum plant growth-promoting traits, i.e., isolates MB-7 and BB-3. Both the isolates were Gram-negative in reaction and positive for the citrate utilization test, and isolate BB-3 was positive for catalase and casein hydrolysis tests, while MB-7 showed negative results for catalase and casein hydrolysis tests.

### Molecular identification of the biofilm-forming bacterial isolates with PGP activities

**Figure 2:**
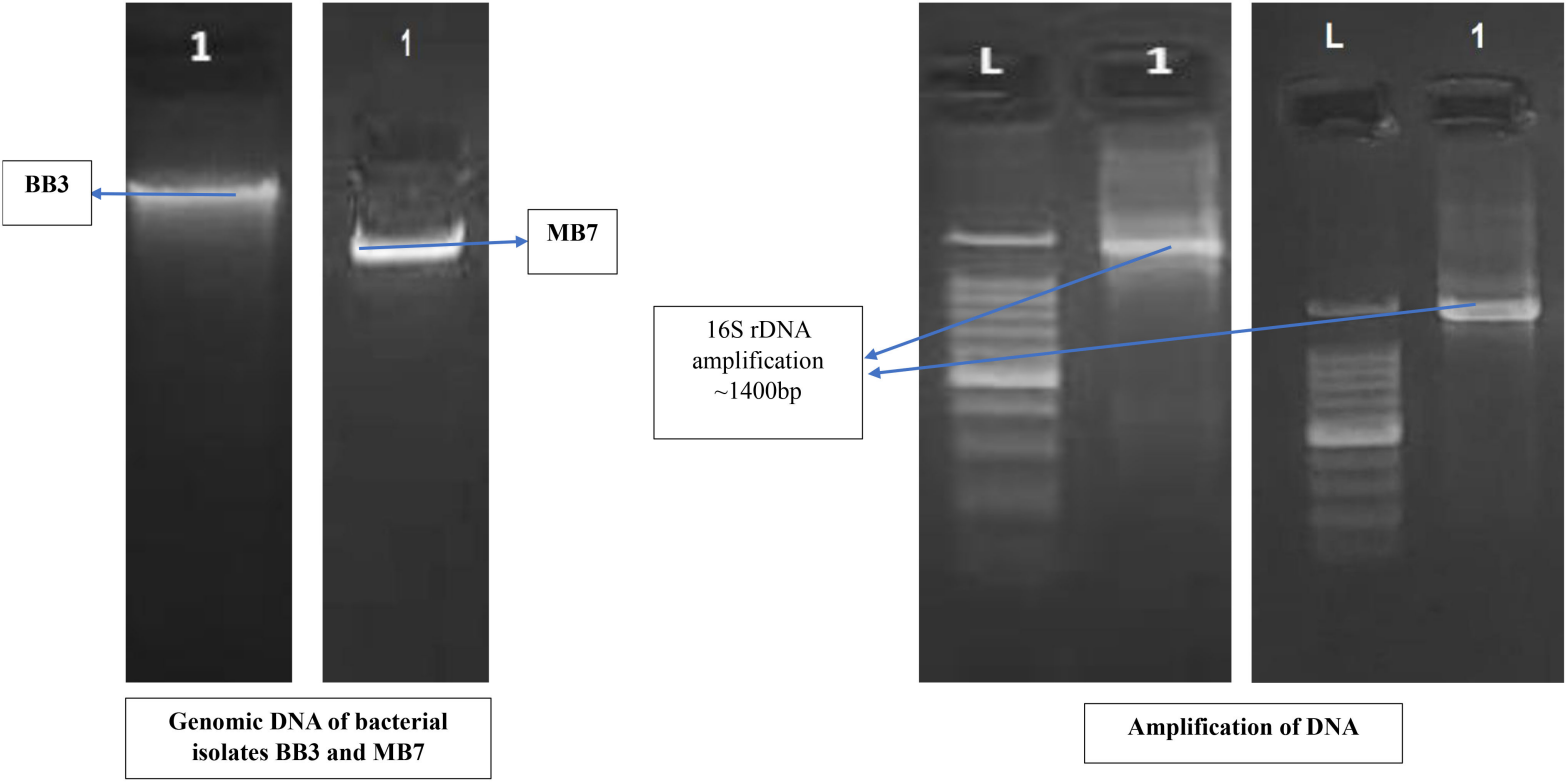
Molecular identification of bacterial isolates based on 16S rDNA amplification.

**Figure 3:**
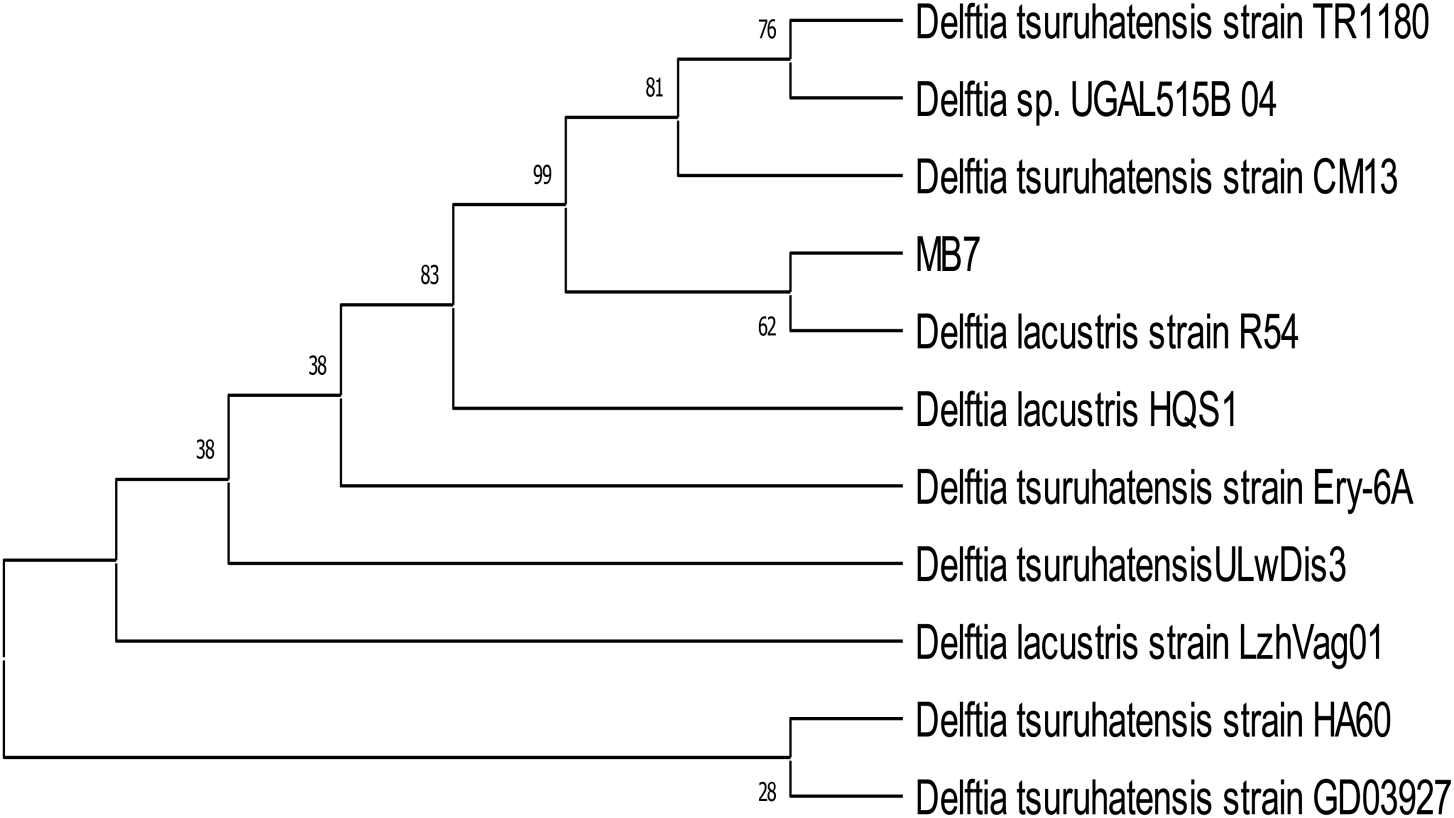
Neighbor-joining tree based on relationship of bacterial isolate MB-7 with the analyzed sequences.

**Figure 4:**
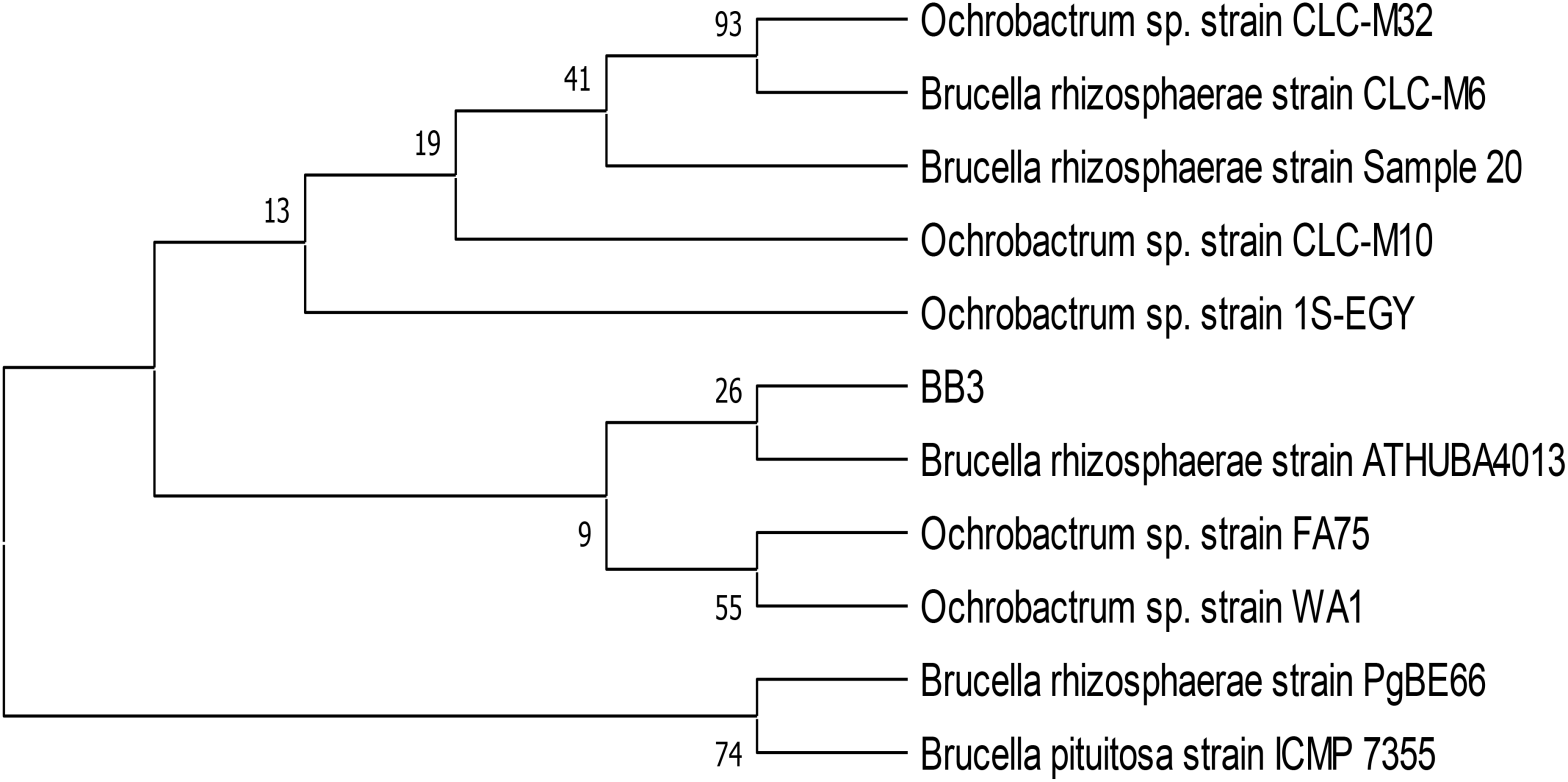
Neighbor-joining tree based on relationship of bacterial isolate BB-3 with the analyzed sequences.

The identification of biofilm-forming PGPR isolates was achieved using PCR amplification of 16S rDNA gene sequences. PGPR isolates BB-3 and MB-7 were chosen for molecular analysis due to their promising biofilm formation and plant growth-promoting capabilities. The selected isolates’ 16S rDNA gene was effectively amplified via PCR, and approximately 1,400 bp of the amplified products was sequenced (**Figure 2**). The BLAST-N comparison of the searched sequences in the National Center for Biotechnology Information (NCBI) nucleotide database revealed 97.18% similarity of MB-7 to *Delftia lacustris* (**Figure 3**) and 99.29% similarity of isolate BB-3 to *Brucella rhizosphaerae* (**Figure 4**).

The isolates’ 16S rDNA sequences were submitted to the NCBI gene bank under the accession numbers PP204779 (release date: January 25, 2024) and OR877951 (release date: November 29, 2023). The quality of both sequences was acceptable. Both samples had good coverage (~1,200+ out of ~1,400 bp amplified), and the quality score was predicted to be high based on the sequencing platform, methods utilized, and high identity percentages in BLAST results. Isolate BB-3 was most closely related to *B. rhizosphaerae* strain ATHUBA4013, and isolate MB-7 grouped most closely with *D. lacustris* strain R54.

### Influence of *B. rhizosphaerae* and *D. lacustris* on vegetative growth of tomato

**Figure 5:**
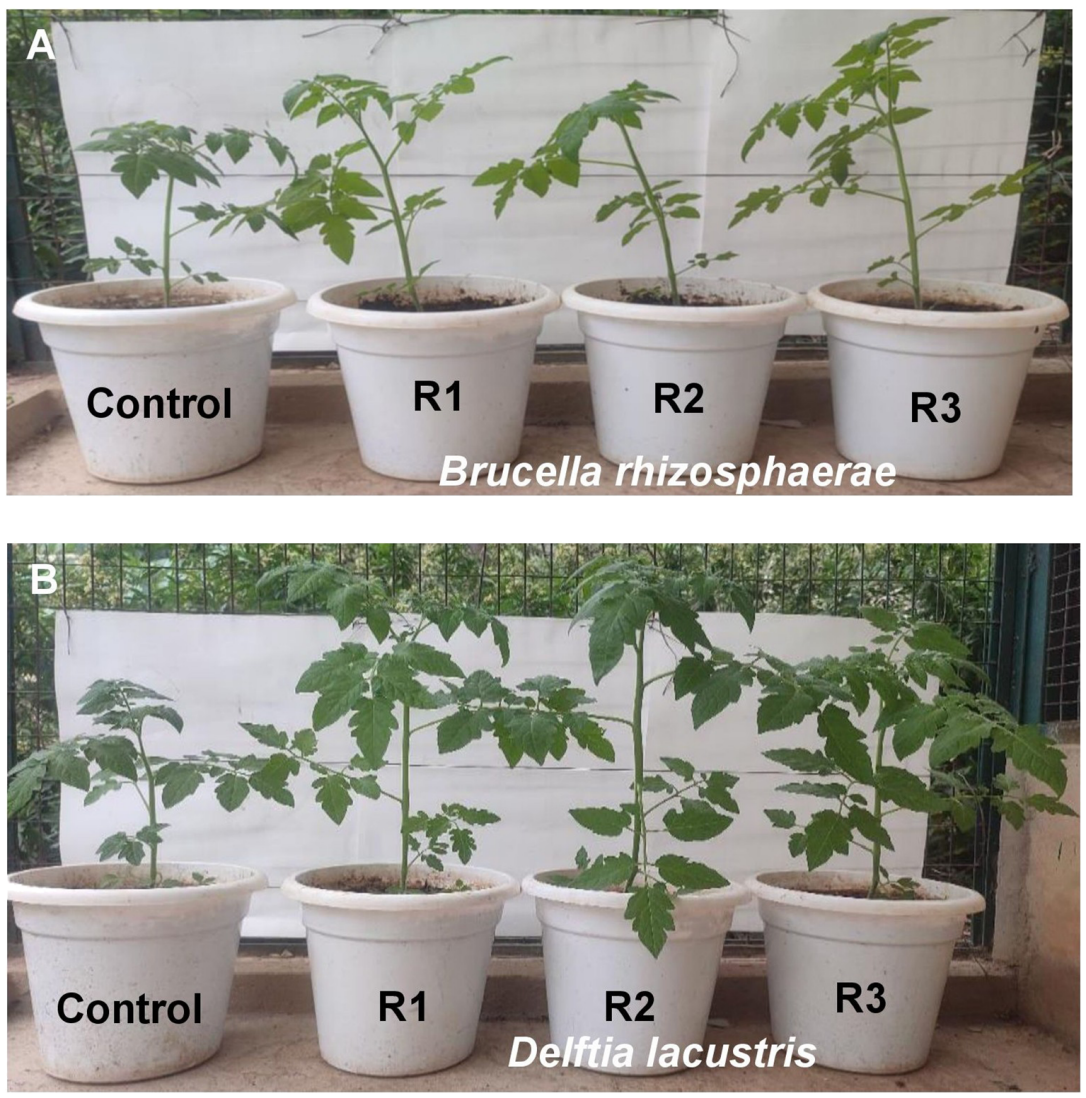
Influence of **(A)** *Brucella rhizosphaerae* and **(B)** *Delftia lacustris* on vegetative growth of tomato. Each treatment was replicated three times. R1, R2, and R3 are replications of the treatment.

**Table 6** and **Figure 5** show that the inoculation of *B. rhizosphaerae* and *D. lacustris* on tomato plants improved plant height by 34.65% and 49.14%, respectively; shoot fresh weight by 30.07% and 32.47%, respectively; and root length by 32.00% and 45.00%, respectively, as compared to control (uninoculated plant).

**Table 6:** Effect of BB-3 and MB-7 inoculation on tomato plant on vegetative growth.

Treatments	Plant height (cm)	Shoot fresh weight (g)	Root length (cm)
Control	35.2 ± 0.15	50.2 ± 0.66	10.0 ± 0.28
BB-3 (*Brucella rhizosphaerae*)	47.4 ± 0.30	65.3 ± 1.65	13.2 ± 0.49
MB-7 (*Delftia lacustris*)	52.5 ± 1.80	66.5 ± 0.24	14.5 ± 0.42
**S.Em.**	0.60	0.59	0.23
**CD _(0.05)_**	2.10	2.06	0.81

Values are the average of triplicates analyzed by ANOVA. ± indicates standard deviation. The significance of the effect was judged with the help of the “F” test. The difference in the mean values was tested using critical difference (CD) at a 5% level of probability. The variance ratio of the test at 0.05% level of significance was further subjected to S.Em.± and CD.

## Discussion

The excessive use of inorganic fertilizers in agriculture has led to environmental degradation and health risks. As a sustainable alternative, biofertilizers, particularly those containing PGPR, offer numerous benefits. Biofilm-forming PGPR strains, such as *Bacillus subtilis* and *Pseudomonas entomophila*, exhibit multiple beneficial traits, including the production of phytohormones, phosphate solubilization, and nitrogen fixation. These bacteria significantly increase plant biomass, root growth, and nutrient uptake in various crops, including lettuce and wheat. In the present study, soil samples were collected from different tomato-growing regions of Himachal Pradesh and analyzed for their physicochemical properties (Shaikh et al., 2018). In a similar study, Swarnalakshmi et al. (2013) reported that key soil macronutrients such as nitrogen, phosphorus, and potassium (NPK) play a crucial role in enhancing microbial activity and are essential for optimal plant growth and productivity. A total of 74 bacteria were isolated from the rhizosphere of tomato plants in different tomato-growing regions of Himachal Pradesh. In a similar study by Zahra et al. (2023), who isolated 11 bacteria from wheat rhizosphere, the bacteria were characterized *in vitro* for plant growth-promoting properties, including IAA production, phosphate solubilization, nitrogen fixation, zinc solubilization, biofilm formation, and cellulase–pectinase production. All isolates showed biofilm formation ability, with WGT4 exhibiting maximum potential.

In the present study, these 74 isolates were screened for various PGP activities. Out of these 74 isolates, 40 bacterial isolates showed positive results for PGP traits out of which all the isolates showed positive results for IAA production with the maximum in MB-7 (89.1 µg/mL) followed by BB-3 (85.1 µg/mL). A similar study by Kumar et al. (2021) showed that *Bradyrhizobium* strains isolated from pigeon pea root nodules showed a wide range of IAA production, from 9.35 to 70.045 μg/mL.

Twenty-eight bacterial isolates demonstrated phosphate solubilization. The maximum phosphate solubilization efficiency was observed in MB-7 (91.2%) followed by isolate BB-3 (86.2%). A similar study by Sharma et al. (2017) showed that *in vitro* evaluation of biocontrol features showed that the highest percent siderophore unit (% SU) was demonstrated by isolate An-14-Mg (71.23% SU) followed by An-15-Mg (70.12% SU), which were significantly at par with each other after 72 h of incubation at 28°C.

Twenty-six isolates showed growth in nitrogen-free Jensen’s media. A study by Patel et al. (2023) demonstrated that a total of 89 isolates were screened for nitrogen fixation using Jensen’s method. This approach involves growing the bacteria in a medium without added nitrogen and then measuring the increase in nitrogen content over time to assess their ability to fix atmospheric nitrogen. The results demonstrated that 54 isolates (60.67%) effectively fixed nitrogen, highlighting their potential as a biofertilizer for enhancing plant growth.

Eighteen bacterial isolates showed HCN production by completely changing the color of filter paper from yellow to dark brown. In a study by Asghar et al. (2023), they screened 26 bacterial strains for HCN production by amending 4.4 g/L glycine in nutrient agar plates. The plates were covered with Whatman filter #1 dipped in the solution of 2% sodium carbonate and 0.5% picric acid. After tightly sealing with parafilm, the cultured agar plates were incubated at 28°C for 4 days; 17 strains produced HCN out of 26 bacterial strains.

Sixteen isolates showed ammonia production by developing a brown-to-yellow color with the addition of Nessler’s reagent. The ammonia production by the PGPR indirectly affects plant growth and development. The PGPR nitrogenous materials of peptones break down into ammonia, which is released into the soil and used by plants as their nutrient source (Backer et al., 2018). A similar study was conducted by Beshah et al. (2023), which reported that all the rhizobial bacterial isolates were able to produce ammonia.

Fifteen isolates showed positive results for siderophore production. The maximum siderophore production efficiency was observed in isolate BB-3 (86.2%) followed by MB-7 (82.6%). Iron is a crucial micronutrient for plants, involved in various physiological processes such as chlorophyll synthesis, electron transport, and nitrogen fixation (Marschner, 2012). Sharma et al. (2024) noted that siderophore-producing microorganisms not only aid in nutrient acquisition but also suppress soil-borne pathogens by outcompeting them for iron. In a study conducted by Soundaryaa and Sharmili (2019), they recorded a maximum siderophore production of 91.74% SU, which was higher than the maximum values of 85% ± 2% for *Pseudomonas fluorescens* PF1 and 78% ± 3% for *B. subtilis* BS3.

A total of 15 bacterial isolates were further screened for biofilm formation using the tissue culture plate method. The maximum average OD value at 595 nm was observed in BB-3 (0.204) followed by MB-7 (0.186). However, the minimum siderophore unit was recorded in SK8 (0.116). Biofilm-forming PGPR, such as *P. entomophila* FAP1, exhibit multiple plant growth-promoting traits and enhanced rhizosphere colonization ability (Ansari and Ahmad, 2018). In a study by Harika et al. (2020), they found that among 92 bacterial isolates from chronic wounds, the standard method tissue culture plate (TCP) detected 64 (69.5%) isolates as strong and eight (8.6%) isolates as moderate biofilm producers, and the remaining 20 (21.7%) isolates were non-biofilm-producing bacteria. The results were recorded at an absorbance of 570 nm where the optical densities were<0.17 for non-biofilm producers, 0.17–0.34 for weak biofilm producers, 0.35–0.68 for moderate biofilm producers, and >0.68 for strong biofilm producers.

The two strong biofilm-forming strains, i.e., BB-3 and MB-7, were further used for biochemical characterization including various tests such as catalase test, citrate utilization, casein hydrolysis, and Gram reaction. Both the isolates were Gram-negative in reaction and positive for the citrate utilization test, and isolate BB-3 was positive for the catalase test and casein hydrolysis, while MB-7 showed negative results for catalase and casein hydrolysis tests. The results are supported by the study of Karagoz et al. (2016), who isolated 188 bacterial strains from different legume plants like clover, sainfoin, and vetch in the Erzurum Province of Turkey; 50 strains were screened for various PGP traits out of which 40 were identified as *Bacillus*, five as *Pseudomonas*, three as *Paenibacillus*, one as *Acinetobacter*, and one as *Brevibacterium*. The catalase test results of all isolates were positive, while oxidase, KOH, and starch hydrolysis test results were variable. Sawant et al. (2022) identified 10 effective *Bacillus* sp. isolated from the rhizosphere of rice and confirmed their identity from positive results of various biochemical tests including citrate utilization, malonate, Voges–Proskauer test, Ortho-Nitrophenyl-β-D-galactopyranoside (ONPG), nitrate reduction, catalase, arginine, sucrose, mannitol, glucose, arabinose, and trehalose.

Isolates BB-3 and MB-7 with significant biofilm-forming and PGPR abilities were sequenced for amplified 16S rDNA gene and identified as *B. rhizosphaerae* and *D. lacustris*, respectively. In a similar study conducted by Rafique et al. (2024), they isolated biofilm-forming plant growth-promoting rhizobacteria from the wheat rhizosphere and molecularly identified one species as *Brucella* sp. through 16S rDNA sequencing. These bacteria create biofilms around plant roots, which improve nutrient uptake, suppress harmful pathogens, and boost plant growth. The ability of PGPR to form biofilms allows them to adhere more effectively to the root surface, providing a stable and effective environment for their growth and activity (Rani et al., 2023).

Two isolates (BB-3 and MB-7) were then applied to tomato plants to evaluate their effect on plant height, shoot fresh weight, and root length. The inoculation of *B. rhizosphaerae* and *D. lacustris* on tomato plants showed values of 47.4 and 52.5 cm, respectively, for plant height; 65.3 and 66.5 g, respectively, for shoot fresh weight; and 13.2 and 14.5 cm, respectively, for root length. The results of the present study are supported by Haque et al. (2020), who reported that with the inoculation of biofilm-forming bacteria, the maximum plant height in tomato plants was attained with treatment T2 (*Pseudomonas azotoformans* ESR4) valued at 58.00 cm as compared to the uninoculated control. Ansari et al. (2021) reported that biofilm-forming strain FAP5-inoculated wheat plants showed a significant increase in shoot fresh and dry weight. The maximum values of shoot fresh and dry weight were 0.214 g and 14.79 mg, respectively, compared to the control. Ricci et al. (2019) reported that with the inoculation of biofilm-forming bacteria, a significant increase of 17.34% with the (Bac HS-Fe) bacterial treatment in the root length of greenhouse tomatoes was observed compared with the uninoculated control (UN HS-Fe).

## Conclusion

This study highlights the potential of biofilm-forming PGPR to improve tomato development under a variety of environmental circumstances. Forty bacterial isolates from tomato rhizosphere soils in Himachal Pradesh were tested for major PGPR properties such as phosphate solubility, nitrogen fixation, IAA, siderophore, HCN, and ammonia production. The TCP technique identified 15 isolates with strong biofilm-forming potential. Two isolates, BB-3 and MB-7, demonstrated robust biofilm development and various PGPR features and were identified as *B. rhizosphaerae* and *D. lacustris*, respectively, using 16S rDNA sequencing. Biofilm production improves bacterial adhesion to roots and protects against abiotic stressors, allowing for continued rhizospheric activity. These PGPR also help with plant defense activity, minimizing the need for chemical inputs. The findings suggest that biofilm-forming PGPR could be used as effective bioinoculants to promote sustainable agriculture and improve crop resilience under a variety of agro-climatic situations.

## Data Availability

The original contributions presented in the study are included in the article/**Supplementary Material**. Further inquiries can be directed to the corresponding authors.
