# Correction: Biofilmed multifarious rhizobacterial isolates of tomato rhizosphere of North-Western Himalayas promote plant growth in tomato

**DOI:** 10.3389/fpls.2025.1658793

**Published:** 2025-07-25

**Authors:** Shubham Kaundal, Neerja Rana, Yashwant Kumar, Saleh S. Alhewairini, Jayanthi Barasarathi, Farah Farhanah Haron, Nazih Y. Rebouh

**Affiliations:** ^1^ Department of Basic Sciences, College of Forestry, Dr. Yashwant Singh Parmar University of Horticulture and Forestry, Solan, India; ^2^ Central Research Institute Kasauli, Kasauli, Solan, India; ^3^ Department of Plant Protection, College of Agriculture and Food, Qassim University, , Qassim, Saudi Arabia; ^4^ Faculty of Health & Life Sciences (FHLS), INTI International University, Nilai, Negeri Sembilan, Malaysia; ^5^ Agrobiodiversity and Environment Research Centre, Malaysian Agricultural Research and Development Institute (MARDI), MARDI Headquarters, Serdang, Selangor, Malaysia; ^6^ Department of Environmental Management, Institute of Environmental Engineering, RUDN University, Moscow, Russia

**Keywords:** ammonia, biofilm-producing, HCN, indole acetic acid, PGPR, P solubilization

In the published article, Affiliation 6 erroneously included “Peoples’ Friendship University of Russia”.

The **Author contributions** statement was erroneously given as “SK: Investigation, Methodology, Writing – original draft. NR: Conceptualization, Project administration, Supervision, Writing – review & editing. YK: Investigation, Methodology, Writing – original draft. SA: Formal Analysis, Funding acquisition, Writing – review & editing. JB: Formal Analysis, Writing – review & editing. FH: Formal Analysis, Writing – review & editing. NYR: Formal Analysis, Funding acquisition, Writing – review & editing”.

The correct **Author contributions** statement is “SK: Investigation, Methodology, Writing – original draft. NR: Conceptualization, Project administration, Supervision, Writing – review & editing. YK: Investigation, Methodology, Writing – original draft. SA: Formal Analysis, Funding acquisition, Writing – review & editing. JB: Formal Analysis, Writing – review & editing. FH: Formal Analysis, Writing – review & editing. NYR: Formal Analysis, Writing – review & editing”.

The original version of this article has been updated.

