# Supplementary material for: Biofilmed multifarious rhizobacterial isolates of tomato rhizosphere of North-Western Himalayas promote plant growth in tomato
Source: Front Plant Sci. 2025 Jul 1;16:1610707. doi: 10.3389/fpls.2025.1610707 (PMC12259571; doi:10.3389/fpls.2025.1610707)
Supplement: Supplementary file 1 [file DataSheet1.docx]

Raw Data:

OD values for biofilm assays

| **Bacterial isolates** | **Average OD value**  **(at 570 nm)** | | |
| --- | --- | --- | --- |
|  | **R1** | **R2** | **R3** |
| **SD-3** | 0.056 | 0.056 | 0.056 |
| **SD-8** | 0.144 | 0.134 | 0.137 |
| **SD-10** | 0.043 | 0.042 | 0.042 |
| **SS-1** | 0.120 | 0.120 | 0.120 |
| **SS-5** | 0.052 | 0.053 | 0.051 |
| **SS-8** | 0.023 | 0.024 | 0.024 |
| **SS-11** | 0.016 | 0.016 | 0.016 |
| **MB-1** | 0.111 | 0.110 | 0.114 |
| **MB-3** | 0.052 | 0.053 | 0.050 |
| **MB-4** | 0.063 | 0.064 | 0.060 |
| **MB-7** | 0.194 | 0.184 | 0.180 |
| **BB-3** | 0.202 | 0.211 | 0.199 |
| **BB-4** | 0.021 | 0.021 | 0.021 |
| **BB-5** | 0.127 | 0.120 | 0.130 |
| **BB-8** | 0.031 | 0.031 | 0.031 |

Absorbance readings for IAA:

| **Bacterial isolates** | **Absorbance value**  **(at 535 nm)** | | |
| --- | --- | --- | --- |
|  | **R1** | **R2** | **R3** |
| **SD-3** | 0.068 | 0.072 | 0.067 |
| **SD-8** | 0.013 | 0.013 | 0.013 |
| **SD-10** | 0.066 | 0.061 | 0.062 |
| **SS-1** | 0.025 | 0.026 | 0.024 |
| **SS-5** | 0.490 | 0.484 | 0.469 |
| **SS-8** | 0.097 | 0.098 | 0.102 |
| **SS-11** | 0.111 | 0.108 | 0.110 |
| **MB-1** | 0.014 | 0.014 | 0.014 |
| **MB-3** | 0.019 | 0.019 | 0.019 |
| **MB-4** | 0.073 | 0.074 | 0.077 |
| **MB-7** | 0.120 | 0.124 | 0.125 |
| **BB-3** | 0.152 | 0.153 | 0.154 |
| **BB-4** | 0.045 | 0.043 | 0.046 |
| **BB-5** | 0.010 | 0.010 | 0.010 |
| **BB-8** | 0.002 | 0.002 | 0.002 |

**Plant Growth Parameters:**

| **Treatments** | **Plant height (cm)** | | | **Shoot fresh weight (g)** | | | **Root length (cm)** | | |
| --- | --- | --- | --- | --- | --- | --- | --- | --- | --- |
|  | **R1** | **R2** | **R3** | **R1** | **R2** | **R3** | **R1** | **R2** | **R3** |
| **Control** | 35.16 | 35.36 | 35.08 | 49.47 | 50.75 | 50.38 | 9.69 | 10.08 | 10.24 |
| **BB-3 (*B.rhizosphaerae*)** | 47.65 | 47.07 | 47.48 | 63.93 | 64.84 | 67.13 | 13.74 | 13.06 | 12.79 |
| **MB-7 (*D.lacustris*)** | 54.50 | 51.00 | 52.00 | 66.30 | 66.43 | 66.77 | 14.15 | 14.96 | 14.38 |

**Preparation of the standard curve (10-100 µg/ml) for IAA Stock solution (100 ppm):**

10 mg of IAA (99.00 %) pure was dissolved in 50 ml distilled water and the final volume was made to 100 ml in a volumetric flask.

| **IAA**  **(ml)** | **Distilled water (ml)** | **Final volume (ml)** | **Salkowski reagent (ml)** | **Ppm**  **(ml)** | **Optical Density (O.D.) at 535 nm** |
| --- | --- | --- | --- | --- | --- |
| 0.0 | 3.0 | 3 | 2 | 0 | 0.00 |
| 0.3 | 2.7 | 3 | 2 | 10 | 0.10 |
| 0.6 | 2.4 | 3 | 2 | 20 | 0.17 |
| 0.9 | 2.1 | 3 | 2 | 30 | 0.25 |
| 1.2 | 1.8 | 3 | 2 | 40 | 0.36 |
| 1.5 | 1.5 | 3 | 2 | 50 | 0.44 |
| 1.8 | 1.2 | 3 | 2 | 60 | 0.56 |
| 2.1 | 0.9 | 3 | 2 | 70 | 0.63 |
| 2.4 | 0.6 | 3 | 2 | 80 | 0.71 |
| 2.7 | 0.3 | 3 | 2 | 90 | 0.79 |
| 3.0 | 0.0 | 3 | 2 | 100 | 0.90 |

**Composition of CAS assay solution**

**2 mM CAS (stock solution):** 0.121 g CAS in 100 ml distilled H_2_O

**1 mM Fe (stock solution):** 1 mM FeCl_3_.6H_2_O in 10 mM HCl

**Piperazine buffer:** Dissolved 4.307 g piperazine in 30 ml distilled water. Added 6.75 ml concentration HCl to bring the pH to 5.6

**Hexadecyl trimethyl ammonium bromide (HDTMA):** Dissolved 0.0219 g HDTMA in 50 ml distilled water in a 100 ml mixing cylinder.

**Preparation of CAS solution**

Mixed 1.5 ml Fe solution with 7.5 ml CAS solution and added to the HDTMA in the mixing cylinder. Added piperazine solution to the mixing cylinder and brought volume up to 100 ml with water.

**Standard curve for indole-3-acetic acid**

**Preparation of the standard curve for P estimation**

0.2195 g KH_2_PO_4_ + Distilled Water = 1 Liter Standard Solution (50 ppm)

| **KH_2_PO_4_**  **(ml)** | **DW**  **(ml)** | **Ammonium molybdate (ml)** | **SnCl_2_**  **(ml)** | Final Volume  25 ml | **Final conc.**  **(ppm)** | **OD at 660 nm** |
| --- | --- | --- | --- | --- | --- | --- |
| 0.0 | 1.0 | 5 | 1 |  | 0 | 0.0 |
| 0.1 | 0.9 | 5 | 1 |  | 5 | 0.08 |
| 0.2 | 0.8 | 5 | 1 |  | 10 | 0.16 |
| 0.3 | 0.7 | 5 | 1 |  | 15 | 0.24 |
| 0.4 | 0.6 | 5 | 1 |  | 20 | 0.30 |
| 0.5 | 0.5 | 5 | 1 |  | 25 | 0.36 |
| 0.6 | 0.4 | 5 | 1 |  | 30 | 0.43 |
| 0.7 | 0.3 | 5 | 1 |  | 35 | 0.49 |
| 0.8 | 0.2 | 5 | 1 |  | 40 | 0.58 |
| 0.9 | 0.1 | 5 | 1 |  | 45 | 0.66 |
| 1.0 | 0.0 | 5 | 1 |  | 50 | 0.74 |

# **Estimation of Phosphorus in sample**

1. 50 ml PVK broth + 10% inoculum (1 O.D. at 660 nm)
2. Incubation under shaking condition
3. Centrifugation
4. 1 ml culture filtrate + distilled water = 25 ml (dilution 25 times)
5. 5 ml of above culture filtrate (step d) + 5ml Ammonium molybdate + 1 ml working solution of SnCl_2_ + Distilled water = final volume to 25 ml (dilution 5 times) O.D. at 660 nm.

Total dilution 25×5 = 125 times

# **Calculations**

Concentration (ppm) from standard curve × 125 (dilution factor) = P-solubilized (ppm). Final concentration calculated after deducting from P-solubilized in control.

**Standard curve for P-estimation**


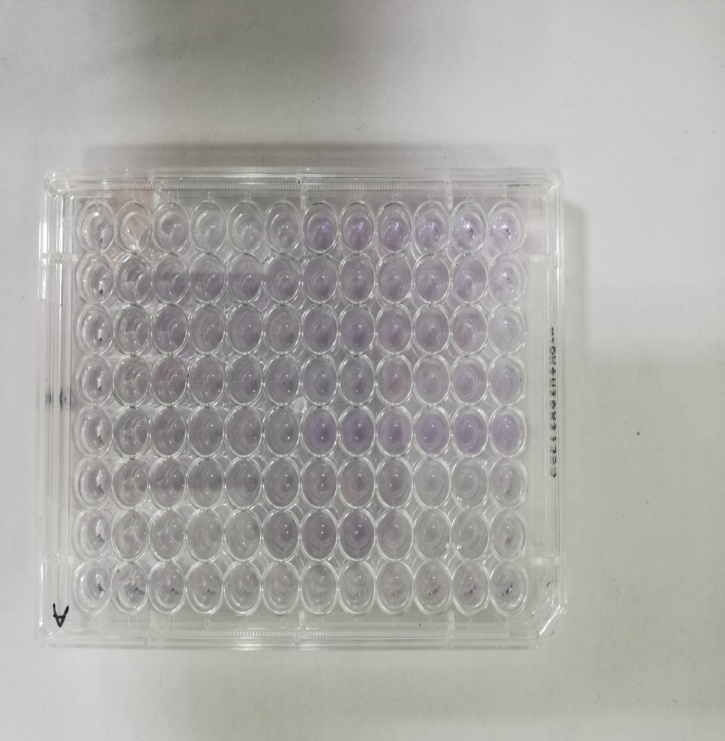


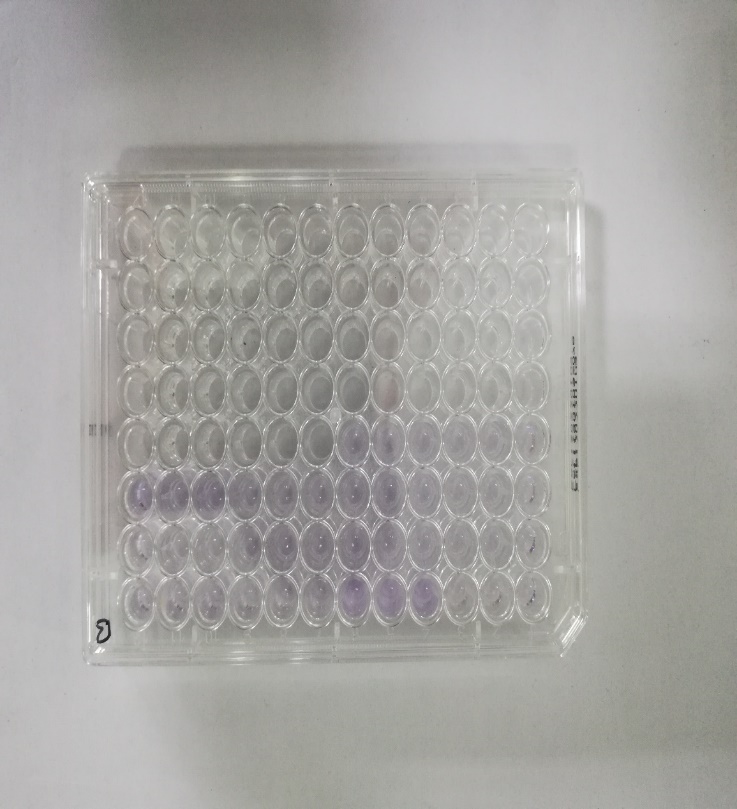


**Biofilm- production by bacterial isolates on Tissue Culture Plate (TCP)**
